# Early Gβγ-GRK2 Inhibition Ameliorates Osteoarthritis Development by Simultaneous Anti-Inflammatory and Chondroprotective Effects

**DOI:** 10.3390/ijms23147933

**Published:** 2022-07-19

**Authors:** Vengadeshprabhu Karuppagounder, William Pinamont, Natalie Yoshioka, Reyad Elbarbary, Fadia Kamal

**Affiliations:** 1 Center for Orthopaedic Research and Translational Science (CORTS), Penn State College of Medicine, 500 University Drive, Hershey, PA 17033-0850, USA; vengadeshprabu@gmail.com (V.K.); wpinamont@pennstatehealth.psu.edu (W.P.); nyoshioka@pennstatehealth.psu.edu (N.Y.); 2 Department of Orthopaedics and Rehabilitation, Penn State College of Medicine, 500 University Drive, Hershey, PA 17033-0850, USA

**Keywords:** osteoarthritis, synovitis, G-protein-coupled receptor kinase 2, therapeutics

## Abstract

The G-protein-coupled receptor kinase 2 (GRK2) is an important regulator of inflammation and pathological macrophage phenotype in a variety of diseases. We hypothesize that Gβγ-GRK2 signaling promotes the early inflammatory response and chondrocyte loss in osteoarthritis (OA). Using the destabilization of the medial meniscus (DMM) model in 12-week-old male C57BL/6 mice, we determined the role of Gβγ-GRK2 signaling in synovitis, macrophage activation, and OA development. We achieved Gβγ-GRK2 inhibition at the time of DMM by administering the Gβγ inhibitor “gallein” and the GRK2 inhibitor “paroxetine” daily, starting from 2 days before DMM surgery, for a duration of 1 or 12 weeks. Synovial and cartilage structural changes were evaluated by histomorphometry, and molecular events and macrophage activation were examined. We studied the direct role of Gβγ-GRK2 in synovitis and macrophage activation in vitro using SW982 and THP1 cells. Continuous Gβγ-GRK2 inhibition initiated at the time of DMM attenuated OA development and decreased chondrocyte loss more effectively than delayed treatment. GRK2 expression and the M1 macrophage phenotype were elevated in the inflamed synovium, while early gallein and paroxetine treatment for 1 and 12 weeks following DMM resulted in their reduction and an upregulated M2 macrophage phenotype. In vitro experiments showed that Gβγ-GRK2 inhibition attenuated synoviocyte inflammation and the M1 phenotype. We show that early Gβγ-GRK2 inhibition is of higher therapeutic efficacy in OA than delayed inhibition, as it prevents OA development by inhibiting the early inflammatory response.

## 1. Introduction

Osteoarthritis (OA) is the major and most common joint disease, characterized by cartilage damage and resulting in joint pain, disability, subchondral bone remodeling with the formation of osteophytes, the degeneration of ligaments and menisci, hypertrophy of the joint capsule, fibrosis of the infrapatellar fat pad, and synovitis [1]. Approximately 19% of the American population is affected by OA, with an especially high incidence rate for adults over 45 years old [2]. OA is also a significant economic burden that results in billions of dollars in annual medical care expenditures in the USA. The global prevalence of knee OA was 16.0% (aged 15 and over) and 22.9% (aged 40 and over), equating to around 654.1 million individuals 40 years or older affected by knee OA in 2020 worldwide [3]. The precise etiology of OA remains unknown, and a cure or treatment for OA is not available.

A large body of evidence suggests that inflammation of the synovium (synovitis) is correlated with the pathogenesis and development of OA [4]. It is well-known that most OA patients have synovial inflammation in all stages of OA [4]. The synovium is formed of an intimal lining layer, which consists of synoviocytes and macrophages, as well as a sub-lining layer composed of connective tissue and blood vessels with few lymphocytes and macrophages [5]. Synovitis is believed to be a precursor of OA [4], as the secretion of cytokines from activated fibroblast-like synoviocytes (FLS) and macrophages results in an inflamed synovium [6,7] and activates chondrocyte inflammation, leading to cartilage damage [8]. Importantly, a recent study reported that synovitis triggers cartilage damage and osteophyte formation in a surgical model of post-traumatic OA (PTOA) [1]. Macrophages are classified according to two major pathways of activation. M1 macrophages are activated by inflammatory stimuli such as interferon (IFN) γ and secrete/trigger numerous proinflammatory cytokines and chemokines [9]. Conversely, M2 macrophages are anti-inflammatory [10].

G-protein-coupled receptors (GPCRs) and their GPCR kinase 2 (GRK2) are important regulators of inflammation [11] and macrophage activation [12,13]. We recently reported that the inhibition of GPCR G-protein beta-gamma (Gβγ)-GRK2 signaling regulates human macrophage activation by promoting their switch to the protective M2 phenotype [10]. In the aforementioned study, we used “gallein” a novel small-molecule Gβγ inhibitor [14,15]. Gallein binds to the GPCR Gβγ subunit and inhibits the recruitment and activation of GRK2. Furthermore, we showed that Gβγ-GRK2 inhibition initiated in progressing OA attenuates cartilage degeneration, promotes anabolic signaling in chondrocytes, and reduces synovitis 12 weeks after the destabilization of the medial meniscus (DMM) [16]. We used paroxetine as a pharmacological GRK2 inhibitor [17,18]. Paroxetine is a selective serotonin reuptake inhibitor (SSRI), commonly used as an FDA-approved antidepressant, that was found to bind GRK2 and inhibit its kinase activity. Several studies showed that both gallein and paroxetine have potent anti-inflammatory effects and regulate the innate immune cell response in various disease models through their Gβγ-GRK2 inhibitory mechanism [19,20,21]. However, the role of Gβγ-GRK2 signaling in the early inflammatory response in OA remains unknown.

We hypothesized that Gβγ-GRK2 signaling is activated in early OA, which promotes inflammation, triggers chondrocyte death, and promotes OA development. Thus, we investigated the therapeutic efficacy of the Gβγ-GRK2 inhibitors gallein and paroxetine in preventing the initial inflammatory response in OA mice using the clinically relevant DMM model in mice. Treatments were initiated at the time of injury and continued for either 1 or 12 weeks following DMM to examine the efficacy from the early to late phases of OA. We determined the effect of treatments on (1) cartilage damage and chondrocyte loss in the late phase of OA (12 weeks following DMM) and (2) GRK2 expression, synovitis, and macrophage activation in both the early inflammatory phase (one week following DMM) and the late phase of OA. Last, we used stable cell lines of human synoviocytes and macrophages to test the direct effects of gallein and paroxetine in vitro. In order to control for the SSRI effect of paroxetine, we used the non-GRK2-inhibiting SSRI fluoxetine. In addition, we used the non-steroidal anti-inflammatory drug (NSAID) indomethacin to control for the reported anti-inflammatory effects of paroxetine and gallein [16,22].

## 2. Results

### 2.1. Early Continuous Gβγ-GRK2 Inhibition Attenuates OA Development in Late-Stage DMM, with Higher Therapeutic Efficacy Than Delayed Treatment

**Figure 1:**
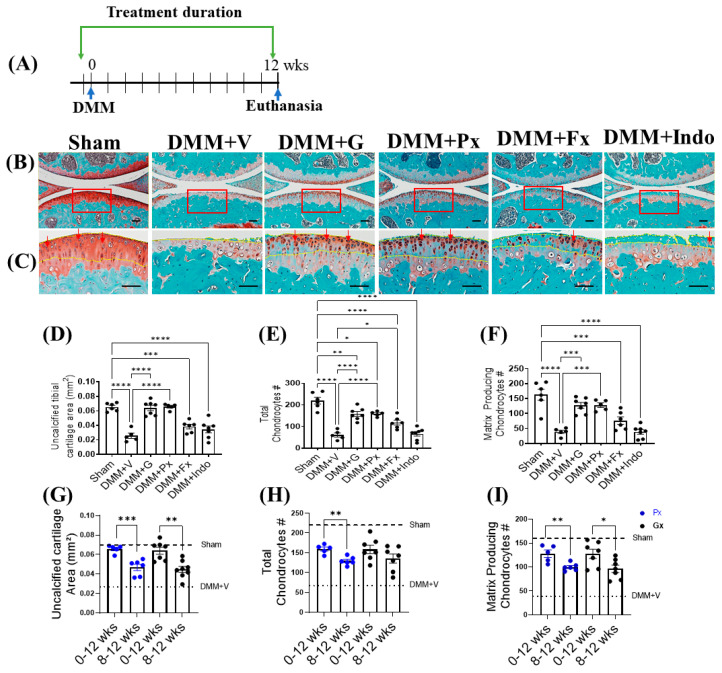
Early continuous Gβγ-GRK2 inhibition prevents PTOA development in mice. (**A**) Schematic representation of timeline of drug treatment after sham (*n* = 5) or DMM surgeries: Two days before surgery, mice started receiving daily intraperitoneal injections of vehicle (V; *n* = 5), gallein (G; 10 mg/kg per day, *n* = 7), paroxetine (Px; 5 mg/kg per day, *n* = 5), fluoxetine (Fx; 5 mg/kg per day, *n* = 6), or indomethacin (Indo; 2.5 mg/kg per day, *n* = 7). All mice were euthanized 12 weeks after surgery. Green arrows denote the duration of drug treatment. (**B**) Representative images of Safranin-O/Fast-Green-stained knee joints; scale bar, 100 µm. (**C**) Magnified images of regions marked by red boxes in (**B**); scale bar, 50 µm. Arrows indicate representative anabolic matrix-producing chondrocytes (dark-red color). Yellow dotted line indicates uncalcified cartilage area in (**C**). Histomorphometric analyses of all treatment groups, showing changes in (**D**) uncalcified cartilage area, (**E**) total chondrocyte number, and (**F**) matrix-producing chondrocyte number. (**G**–**I**) Histomorphometric analyses comparing early and delayed continuous gallein (black color points) and paroxetine (blue color points) treatment groups (0–12 weeks and 8–12 weeks post-DMM), showing changes in (**G**) uncalcified cartilage area, (**H**) total chondrocyte number, and (**I**) matrix-producing chondrocyte number. * *p* < 0.05, ** *p* < 0.01, *** *p* < 0.001, and **** *p* < 0.0001 using one-way ANOVA. * *p* < 0.05, ** *p* < 0.01, and *** *p* < 0.001 0–12 weeks vs. 8–12 weeks treatment groups using Welch’s *t*-test. Values are expressed as means ± SEM.

Twelve weeks after DMM, vehicle-treated DMM mice had extensive cartilage degeneration and chondrocyte loss, as reflected in the significant reduction in the uncalcified cartilage area and the total and anabolic chondrocyte numbers (Figure 1A–C). Continuous Gβγ-GRK2 inhibition by gallein and paroxetine, initiated at the time of DMM and continued for 12 weeks (Figure 1A–C), decreased cartilage degeneration, as evidenced by the preserved uncalcified cartilage area (Figure 1D) and the total (Figure 1E) and anabolic chondrocytes (dark-red color) (Figure 1C,F) at levels similar to those of the sham mice. While fluoxetine partially rescued the total number of chondrocytes, indomethacin failed to confer any protection in the cartilage of DMM mice. These results suggest that continuous Gβγ-GRK2 inhibition by gallein and paroxetine prevents cartilage degeneration, chondrocyte loss, and OA development in late-stage PTOA.

We compared the chondroprotective effect of early continuous treatment with gallein and paroxetine (from the time of DMM to week 12; 0–12) to our published data with delayed treatment (from week 8 following DMM to week 12; 8–12) [16]. The early treatment was more effective, as evidenced by the higher area of the uncalcified cartilage and the numbers of total and anabolic chondrocytes (Figure 1G–I).

### 2.2. Early Gβγ-GRK2 Inhibition Attenuates Chondrocyte Apoptosis and Cytochrome C in the Acute Inflammatory Phase of DMM

**Figure 2:**
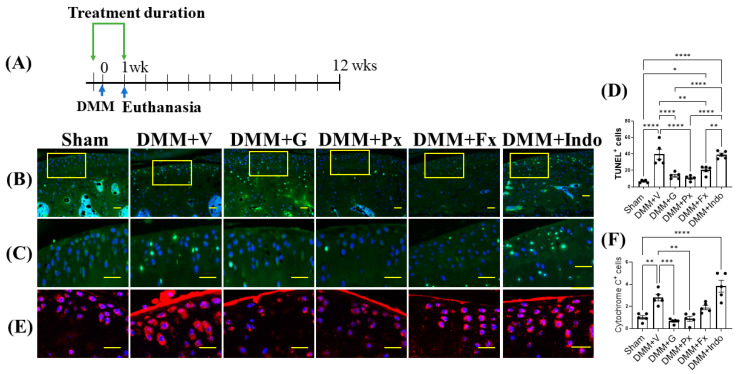
Early continuous Gβγ-GRK2 inhibition reduces chondrocyte apoptosis and cytochrome C expression in mouse early PTOA cartilage. (**A**) Schematic representation of timeline of drug treatment after sham (*n* = 5) or DMM surgeries. Two days before surgery, mice started receiving daily intraperitoneal injections of vehicle (V; *n* = 10), gallein (G; 10 mg/kg per day, *n* = 10), paroxetine (Px; 5 mg/kg per day, *n* = 7), fluoxetine (Fx; 5 mg/kg per day, *n* = 9), or indomethacin (Indo; 2.5 mg/kg per day, *n* = 9). All mice were euthanized 1 week after surgery. Green arrows denote the duration of drug treatment. Representative images showing fluorescent staining in the anterior femoral synovium: (**B**) TUNEL staining of apoptotic cells; scale bar, 50 µm. (**C**) Magnified images of regions marked by yellow boxes in (**B**); scale bar, 10 µm. (**D**) Quantification of TUNEL-positive cells. (**E**) IF staining of cytochrome C (red); scale bar, 10 µm. (**F**) Quantification of the percentage of cytochrome-C-positive cells. DAPI (blue) stains nuclei. *n* = 5 per group; * *p* < 0.05, ** *p* < 0.01, *** *p* < 0.001, and **** *p* < 0.0001 in 1 week treatment groups using one-way ANOVA. Values are expressed as means ± SEM.

Next, we investigated the possible mechanism behind the higher efficacy of early Gβγ-GRK2 inhibition in OA. We determined the acute effect of early treatment on chondrocyte death in the early inflammatory phase (Figure 2A) by examining TUNEL staining 1 week after DMM. The number of apoptotic chondrocytes was increased in the vehicle-treated group relative to the sham group and was reduced by the gallein, paroxetine, and fluoxetine treatments but not by indomethacin (Figure 2B–D). Finally, we quantified the acute changes in chondrocyte cytochrome C (Cyt c) expression by IF staining in the cartilage 1 week after DMM. Cyt c expression was elevated in the vehicle-treated mice and was reduced by gallein and paroxetine but not by fluoxetine or indomethacin (Figure 2E,F).

### 2.3. Elevated Synovial GRK2 Expression in the Early Inflammatory Phase following DMM Is Reduced by Gβγ-GRK2 Inhibition

**Figure 3:**
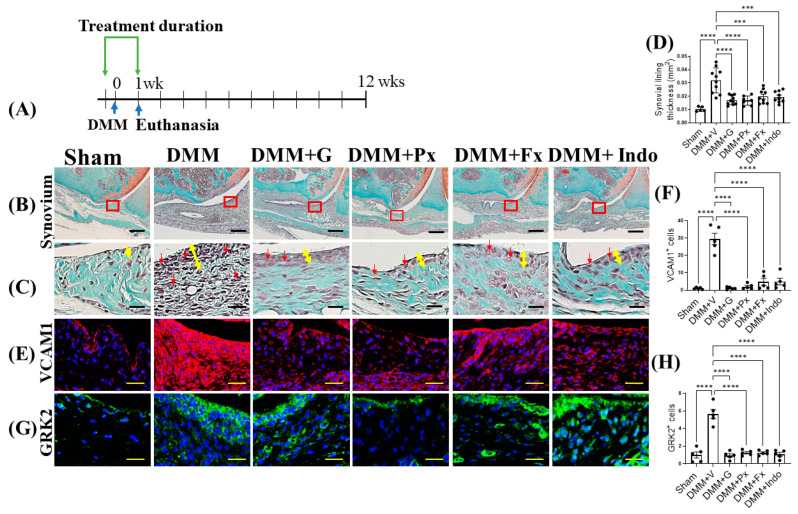
Early continuous Gβγ-GRK2 inhibition attenuates synovitis in early-stage PTOA. (**A**) Schematic representation of timeline of drug treatment after sham (*n* = 5) or DMM surgeries. Two days before surgery, mice started receiving daily intraperitoneal injections of vehicle (V; *n* = 10), gallein (G; 10 mg/kg per day, *n* = 10), paroxetine (Px; 5 mg/kg per day, *n* = 7), fluoxetine (Fx; 5 mg/kg per day, *n* = 9), or indomethacin (Indo; 2.5 mg/kg per day, *n* = 9). All mice were euthanized 1 week after surgery. Green arrows denote the duration of treatment. (**B**) Representative images of Safranin-O/Fast Green staining of the anterior femoral synovial region of mouse knee sections 1 week after DMM or sham surgery; scale bar, 50 µm. (**C**) Magnified images of regions indicated in red boxes in (**B**); scale bar, 10 µm. Yellow double-headed arrows depict synovial lining thickness, and red arrows indicate synovial inflammatory cells. (**D**) Quantification of anterior femoral synovial membrane thickness. Representative IF staining images showing (**E**) VCAM1 (red) and (**G**) GRK2 (green) in the anterior femoral synovium. DAPI (blue) stains nuclei; scale bar, 50 µm. Quantification of the percentage of positive cells expressing (**F**) VCAM1 and (**H**) GRK2. *n* = 5 per group; *** *p* < 0.001 and **** *p* < 0.0001 vs. DMM + V group using one-way ANOVA. Values are expressed as means ± SEM.

We then explored synovial changes in the early and late stages of OA. In early-stage OA, one week after DMM (Figure 3A), the synovium of vehicle-treated mice was thicker than that of the sham-operated mice, indicating synovitis with increased inflammatory cell infiltration (red arrows) (Figure 3B–D). This was corroborated by increased vascular cell adhesion protein (VCAM) 1 expression in the vehicle-treated DMM mice, with a parallel increase in GRK2 expression. The Gβγ-GRK2 inhibition by gallein and paroxetine reduced synovial thickness and inflammatory cell infiltration (Figure 3B–D) and VCAM1 and GRK2 expression (Figure 3E–H). Treatment with fluoxetine and indomethacin also attenuated synovial thickness, inflammatory cell infiltration, and VCAM1 and GRK2 expression in DMM mice when compared with vehicle-treated DMM mice (Figure 3B–H). These results suggest that Gβγ-GRK2 signaling plays a major role in the initiation of the inflammatory response and synovitis following traumatic injury in OA, while its inhibition attenuates synovitis in early OA.

**Figure 4:**
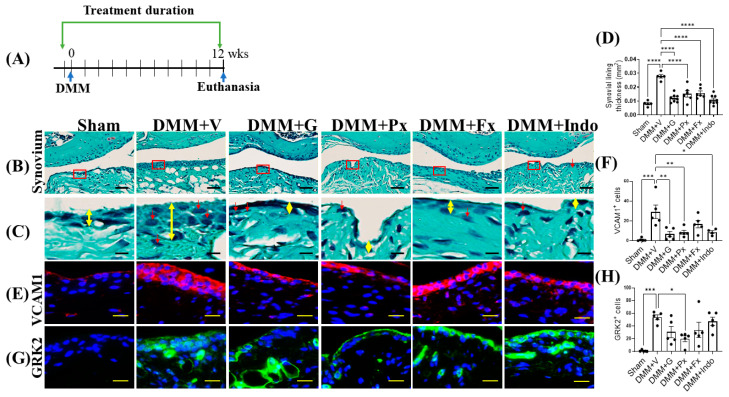
Early continuous Gβγ-GRK2 inhibition attenuates synovitis in late-stage PTOA. (**A**) Schematic representation of timeline of drug treatment after sham (*n* = 5) or DMM surgeries: Two days before surgery, mice started receiving daily intraperitoneal injections of vehicle (V; *n* = 5), gallein (G; 10 mg/kg per day, *n* = 7), paroxetine (Px; 5 mg/kg per day, *n* = 5), fluoxetine (Fx; 5 mg/kg per day, *n* = 6), or indomethacin (Indo; 2.5 mg/kg per day, *n* = 7). All mice were euthanized 12 weeks after surgery. Green arrows denote the duration of drug treatment. (**B**) Representative images of Safranin-O/Fast Green staining of the anterior femoral synovial region of mouse knee sections 12 weeks after DMM or sham surgery; scale bar, 50 µm. (**C**) Magnified images of regions indicated in red boxes in (**B**); scale bar, 10 µm. Yellow double-headed arrows depict synovial lining thickness, and red arrows indicate synovial inflammatory cells. (**D**) Quantification of anterior femoral synovial membrane thickness. Representative IF staining images in the anterior femoral synovium showing (**E**) VCAM1 (red) and (**G**) GRK2 (green). DAPI (blue) stains nuclei; scale bar, 50 µm. Quantification of the percentage of positive cells expressing (**F**) VCAM1 and (**H**) GRK2. *n* = 5 per group; * *p* < 0.05, ** *p* < 0.01, *** *p* < 0.001, and **** *p* < 0.0001 vs. DMM + V group using one-way ANOVA; values are expressed as means ± SEM.

In late-stage OA, twelve weeks after DMM (Figure 4A), vehicle-treated DMM mice continued to have a thicker synovium with higher levels of inflammatory cells (red arrows) relative to the sham-operated mice, while the continuous treatment with gallein and paroxetine attenuated these pathological changes (Figure 4B–D). Interestingly, this anti-inflammatory effect was comparable to that of indomethacin (NSAID), which is widely used for pain management in OA patients, and that of the non-GRK2-inhibiting SSRI fluoxetine (Figure 4B–D). On the cellular level, the synovium of vehicle-treated DMM mice continued to show high levels of expression of both synoviocyte inflammatory markers, VCAM1 and GRK2, while both gallein and paroxetine treatments reduced their expression (Figure 4E–H). While VCAM1 expression was reduced by indomethacin and not by fluoxetine, neither indomethacin nor fluoxetine reduced GRK2 expression in the synovium of mice 12 weeks after DMM (Figure 4E–H). These findings suggest that Gβγ-GRK2 signaling maintains inflammation and synovitis following traumatic injury in OA, while its continuous inhibition attenuates synovitis in late OA.

### 2.4. Gβγ-GRK2 Inhibition Promotes M2 over M1 Macrophage Phenotype in Early and Late DMM

**Figure 5:**
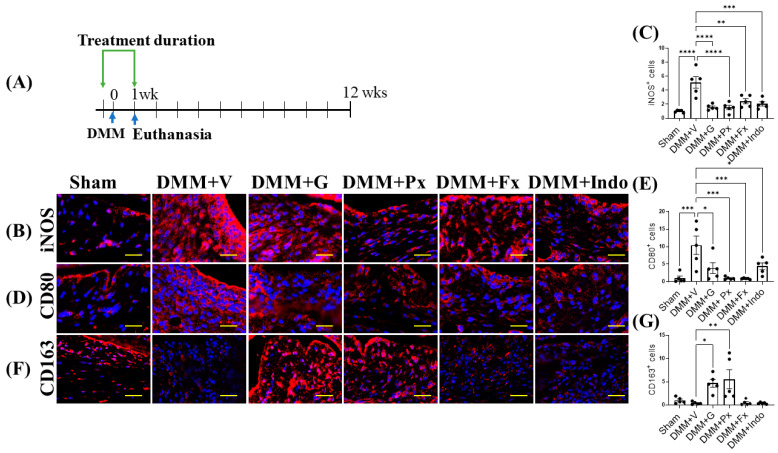
Early continuous Gβγ-GRK2 inhibition switches synovial macrophage phenotype to M2 in early-stage PTOA. (**A**) Schematic representation of timeline of drug treatment after sham (*n* = 5) or DMM surgeries. Two days before surgery, mice started receiving daily intraperitoneal injections of vehicle (V; *n* = 10), gallein (G; 10 mg/kg per day, *n* = 10), paroxetine (Px; 5 mg/kg per day, *n* = 7), fluoxetine (Fx; 5 mg/kg per day, *n* = 9), or indomethacin (Indo; 2.5 mg/kg per day, *n* = 9). All mice were euthanized 1 week after surgery. Green arrows denote the duration of drug treatment. Representative images of IF staining of (M1 markers) (**B**) iNOS, (**D**) CD80, and (**F**) CD163 (M2 marker) in the in the anterior femoral synovium of sham and DMM mouse knee sections 1 week after DMM or sham surgery. Target proteins are represented in red. DAPI (blue) stains nuclei; scale bar, 50 µm. Quantification of the percentage of positive cells expressing (**C**) iNOS, (**E**) CD80, and (**G**) CD163. *n* = 5 per group; * *p* < 0.05, ** *p* < 0.01, *** *p* < 0.001, and **** *p* < 0.0001 vs. DMM + V group using one-way ANOVA. Values are expressed as means ± SEM.

One week after DMM (Figure 5A), the expression of the M1 macrophage phenotype markers inducible nitric oxide synthase (iNOS) and cluster of differentiation (CD) 80 were elevated (Figure 5B–E), while the macrophage M2 marker CD163 trended lower in the synovium of vehicle-treated DMM mice (Figure 5F,G), indicating increased macrophage activation, infiltration, and M1 phenotype. Treatment with gallein and paroxetine attenuated iNOS and CD80 expression, while increasing CD163 expression (Figure 5B–G). Both the fluoxetine and indomethacin treatments reduced iNOS and CD80 expression but had no effect on CD163 expression (Figure 5B–G). Altogether, these results suggest that gallein and paroxetine reverse the M1 macrophage phenotype in favor of the M2 phenotype in the early phase of DMM. On the other hand, as fluoxetine and indomethacin only inhibit M1 markers, their anti-inflammatory effects are less potent than gallein and paroxetine.

**Figure 6:**
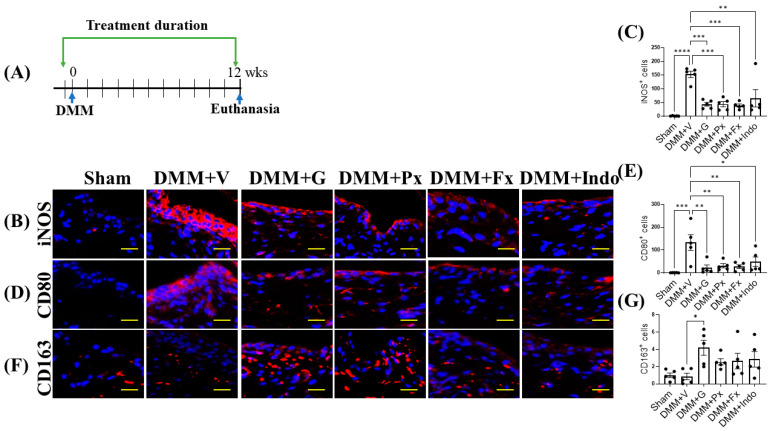
Early continuous Gβγ-GRK2 inhibition switches synovial macrophage phenotype to M2 in late-stage PTOA. (**A**) Schematic representation of timeline of drug treatment after sham (*n* = 5) or DMM surgeries. Two days before surgery, mice started receiving daily intraperitoneal injections of vehicle (V; *n* = 5), gallein (G; 10 mg/kg per day, *n* = 7), paroxetine (Px; 5 mg/kg per day, *n* = 5), fluoxetine (Fx; 5 mg/kg per day, *n* = 6), or indomethacin (Indo; 2.5 mg/kg per day, *n* = 7). All mice were euthanized 12 weeks after surgery. Green arrows denote the duration of drug treatment. Representative images of IF staining of (M1 markers) (**B**) iNOS, (**D**) CD80, and (**F**) CD163 (M2 marker) in the in the anterior femoral synovium of sham and DMM mouse knee sections 12 weeks after DMM or sham surgery. Target proteins are represented in red. DAPI (blue) stains nuclei; scale bar, 50 µm. Quantification of the percentage of positive cells expressing (**C**) iNOS, (**E**) CD80, and (**G**) CD163. *n* = 5 per group; * *p* < 0.05, ** *p* < 0.01, *** *p* < 0.001, and **** *p* < 0.0001 vs. DMM + V group using one-way ANOVA; values are expressed as means ± SEM.

Twelve weeks after DMM (Figure 6A), iNOS and CD80 expression remained at levels higher than those of sham-operated mice (Figure 6B–E). All drug treatments reduced iNOS and CD80 expression, but only gallein and paroxetine increased CD163 expression (Figure 6B–G). These results indicate that the Gβγ-GRK2 inhibitors gallein and paroxetine inhibit synovial inflammation and promote the switch from the M1 to the M2 macrophage phenotype throughout the DMM disease course from early- to late-stage PTOA.

### 2.5. Gβγ-GRK2 Inhibition Ameliorates Human Synoviocytes and M1 Macrophage Inflammatory Differentiation In Vitro

**Figure 7:**
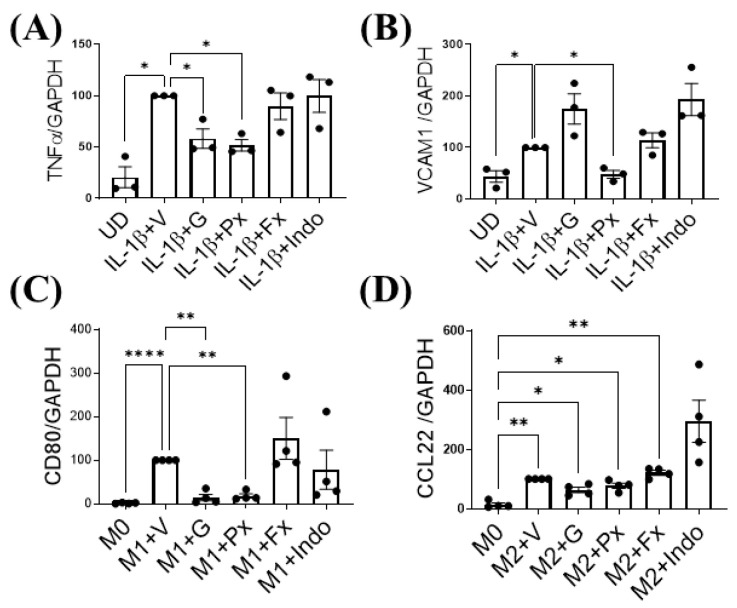
Gβγ-GRK2 inhibition ameliorates synoviocyte inflammation and M1 macrophage differentiation in human cells. Changes in mRNA gene expression of synoviocyte inflammatory markers (**A**) TNFα and (**B**) VCAM1 in human synoviocytes (FLS-differentiated SW982 cells and undifferentiated cells (UD)). IL-1β-stimulated SW982 cells were treated with vehicle (IL-1β + V) gallein (1β + G), paroxetine (1β + Px), fluoxetine (1β + Fx), and indomethacin (1β + Indo). Changes in mRNA gene expression of macrophage phenotype markers (**C**) CD80 and (**D**) CCL22 in human macrophage cells (M1- and M2-differentiated THP1 cells). Macrophage cells differentiated into M1 or M2 macrophage phenotypes and resting macrophages (M0) were treated with vehicle (M1/M2 + V), gallein (M1/M2 + G), paroxetine (M1/M2 + Px), fluoxetine (M1/M2 + Fx), and indomethacin (M1/M2 + Indo). RT-qPCR from *n* = 3 experimental replicates. Data are normalized to expression of GAPDH. * *p* < 0.05 vs. IL-1β + V group; ** *p* < 0.01 and **** *p* < 0.0001 vs. M1 + V group; * *p* < 0.05 and ** *p* < 0.01 vs. M0 + V group using one-way ANOVA. Values are expressed as means ± SEM.

We determined the direct effect of Gβγ-GRK2 inhibition on both macrophage and synoviocyte inflammatory signaling by treating FLS-differentiated human SW982 cells and M1-differentiated human THP1 cells with gallein and paroxetine in vitro. Treatment with gallein and paroxetine inhibited the mRNA expression of the synoviocyte inflammatory marker tumor necrosis factor (TNF) α (Figure 7A). On the other hand, only paroxetine inhibited VCAM1 expression, with gallein treatment being statistically insignificant (Figure 7B). Further, paroxetine and gallein attenuated mRNA expression of M1 macrophage marker CD80 (Figure 7C) and upregulated M2 macrophage marker C-C motif chemokine (CCL) 22 (Figure 7D). In contrast, indomethacin and fluoxetine treatment had no effect on inflammatory gene expression in either synoviocytes or macrophages **(**Figure 7A–D). Therefore, these results show that the Gβγ-GRK2 pharmacological inhibition by gallein and paroxetine directly attenuates the inflammatory phenotypes of synoviocytes and macrophages under inflammatory conditions in vitro, which resemble PTOA.

## 3. Discussion

In the current study, we investigated the therapeutic efficacy of Gβγ-GRK2 inhibition in attenuating inflammation and preventing OA development when administered at the time of injury in DMM mice. We recently reported that Gβγ-GRK2 inhibition attenuates cartilage degeneration and synovitis in ongoing PTOA when administered from week 8 to week 12 following DMM [16]. Here, we found that Gβγ-GRK2 inhibition, by gallein and paroxetine treatment at the time of injury, exerted a strong anti-inflammatory effect extending from the early stages to the late stages of DMM. This early intervention protects the chondrocytes from apoptosis in the early phase of OA, thereby preserving the articular cartilage and ameliorating OA development.

Osteoarthritis is the most common joint disease, leading to severely affected quality of life and mobility due to degenerative joint changes, including cartilage degeneration, subchondral bone remodeling, osteophytes formation, synovitis, the degeneration of ligaments and menisci of the knee, and hypertrophy of the joint capsule [1].

A large body of evidence suggests that inflammation of the synovium (synovitis) correlates with the pathogenesis and development of OA [4,23,24]. We hypothesized that early Gβγ-GRK2 inhibition can impart protection to prevent or decelerate OA development in the early inflammatory phase following DMM due to their outlined anti-inflammatory effects [10,16]. Therefore, we compared the chondroprotective and chondroregenerative effects of gallein and paroxetine treatment initiated in the early-stages (at the time of injury) to our recently reported results with delayed treatment initiation (8 weeks following DMM) [16]. The early treatment was more chondroprotective and matrix-regenerative, where the area of uncalcified cartilage was maintained at levels close to those of sham mice, indicating attenuated OA development. To determine the possible mechanism behind this effect, we investigated changes in chondrocyte apoptosis one week after DMM. In early OA, inflammatory synoviocytes and macrophages secrete cytokines that lead to cartilage damage and OA development [1,8], and apoptosis plays a major role in this process [25]. The excess production of reactive oxygen species and inflammation triggers [26] programmed cell death [27], leading to cartilage degradation and OA development [28]. Moreover, plenty of experimental and clinical data recapitulated the release of Cyt c from dying cells, either due to apoptosis or necrosis [29], which in turn induces a series of biochemical reactions that result in subsequent cell death [30]. In DMM mice, we found increased chondrocyte apoptosis one week after injury, with a parallel increase in high Cyt c expression. Both apoptosis and Cyt c expression were reduced following gallein and paroxetine treatment. Interestingly, fluoxetine-treated mice also had reduced chondrocyte apoptosis. Although their Cyt c expression was not less than that of the vehicle-treated DMM group, it was not significantly higher than that of the sham mice; similar results were previously reported in vitro [31]. On the other hand, indomethacin treatment increased chondrocyte apoptosis and Cyt c expression, also previously reported in vitro [32,33]. Accordingly, these results suggest that gallein, paroxetine, and fluoxetine protect articular chondrocytes from apoptosis following DMM, with the possible involvement of mitochondrial dysfunction. However, further studies are warranted to dissect the exact molecular mechanism involved. From a therapeutic point of view, an earlier initiation of treatment with gallein and paroxetine can reduce chondrocyte apoptosis and loss, which in combination with their anabolic effect [16], attenuates OA development and progression, thereby providing a superior therapeutic benefit in OA.

We then explored the effect of continuous Gβγ-GRK2 inhibition on early- to late-stage inflammation in OA. One week after DMM, mice had significant synovitis, which declined with time but remained at levels higher than in sham mice 12 weeks after DMM. Importantly, at both stages, synovitis was associated with high levels of GRK2 expression in the synovium. Continuous Gβγ-GRK2 inhibition with gallein and paroxetine from the time of injury to week 12 exerted an anti-inflammatory effect, as evidenced by reduced synovial lining thickness, inflammatory cell infiltration, and VCAM1 expression, comparable to that of indomethacin, an NSAID that is widely used for pain management in OA patients. This agrees with the reported effects of targeted GRK2 kinase domain inhibition to reverse synoviocyte dysfunction and ameliorate collagen-induced arthritis in a rat model [34]. Fluoxetine, an SSRI that lacks GRK2-inhibiting properties, also exerted anti-inflammatory effects in DMM mice, as previously reported in other models [35,36]. The anti-inflammatory effects of all treatments were associated with reduced GRK2 expression in the synovium, which shows a direct correlation between GRK2 inhibition and attenuated synovitis, regardless of the anti-inflammatory drug mechanism. Notably, neither fluoxetine nor indomethacin conferred protection in the cartilage of OA mice, unlike gallein and paroxetine, which might be attributed to the different anti-inflammatory mechanisms involved, in addition to the direct chondroprotective effect of gallein and paroxetine on chondrocytes that we recently reported [16] and their protective effect against mitochondrial dysfunction and apoptosis, as discussed above.

To investigate the possible protective anti-inflammatory mechanisms of early Gβγ-GRK2 inhibition, we investigated macrophage activation and phenotype in DMM mice receiving different treatments. Various immune cells are responsible for the development of synovitis in OA patients. Of these cells, macrophages are a highly abundant population of the infiltrate in synovial tissues of OA patients [37], where they represent approximately 65% of the infiltrating immune cells [38]. Macrophages are classified according to two major subtypes: proinflammatory M1 and anti-inflammatory M2. The deleterious effects of M1 macrophages on joint inflammation and cartilage degeneration have been reported in OA [39,40]. The chondroprotective effects of M2 macrophages were also reported in an OA mouse model [1], while the depletion of both M1 and M2 macrophages increases inflammation and does not attenuate OA [41]. Furthermore, a macrophage phenotypic switch from M2 to M1 is a driving pathological mechanism in experimental OA [1]. Importantly, GRK2 is known to regulate pathologic macrophage activation [12,13], and we have reported that Gβγ-GRK2 inhibition promotes the switch to the protective M2 phenotype in human macrophages in vitro [10]. In DMM mice, we found increased M1 macrophages in the synovium 1 week after injury, which continued until week 12. Gallein and paroxetine attenuated M1 and promoted M2 macrophages in the synovium of OA mice 1 week after DMM and continued until week 12. On the other hand, fluoxetine and indomethacin attenuated M1 macrophages but had no effect on M2 macrophages. This effect by indomethacin has been reported in different tissues and diseases [42,43]. Altogether, this suggests that Gβγ-GRK2 inhibition exerts superior anti-inflammatory effects in OA by attenuating the proinflammatory M1 macrophages and promotes healing by increasing the anti-inflammatory M2 macrophages, in contrast to fluoxetine and indomethacin, which inhibit both M1 and M2 macrophages.

Finally, we confirmed a direct anti-inflammatory effect of gallein and paroxetine on synoviocyte inflammation and macrophages in vitro. Both gallein and paroxetine attenuated the SW982 human synoviocyte FLS inflammatory phenotype, while fluoxetine and indomethacin had no effect. In THP1 human macrophages, gallein and paroxetine suppressed M1 but not M2 macrophage marker expression. Interestingly, in opposition to their in vivo effect, fluoxetine and indomethacin did not inhibit M1 macrophage marker expression. Altogether, this supports our in vivo results where Gβγ-GRK2 inhibition by gallein and paroxetine favored the M2 over the M1 macrophage phenotype, thus promoting healing in OA.

An overwhelming majority of people with OA have synovitis. Synovial inflammation is present in all the stages of OA and is associated with the initiation and progression of cartilage degeneration [4]. We recently reported the chondroprotective and matrix-regenerative effects of the Gβγ-GRK2 inhibitors gallein and paroxetine in DMM mice when treatment was initiated in progressing OA to attenuate OA progression [16]. In the current study, we demonstrate the protective effects of treatment with the Gβγ-GRK2 inhibitors gallein and paroxetine in DMM-induced PTOA when treatment was initiated at the time of injury to attenuate OA development through early intervention. The mechanisms behind this effect include the promotion of a pro-healing, anti-inflammatory signaling cascade in the synovium, the inhibition of chondrocyte apoptosis, and increased anabolic signaling, as we recently reported [16]. These results present early Gβγ-GRK2 inhibition as a protective therapeutic approach in PTOA.

This study has some limitations. The exact mechanisms involved in the effects of Gβγ-GRK2 inhibition in DMM mice, particularly on the cartilage and synovium, were not investigated in this study, mainly due to the technical limitation of the small size of the mouse joint. Studies using larger animals that enable protein and RNA isolation will shed light on these mechanisms. In the in vitro experiments, we used the SW982 cell line, which was derived from a synovial sarcoma. Further studies using human primary synoviocytes will increase the translational application of this pathway in the human synovium. Finally, we showed that Gβγ-GRK2 inhibition favored an M2 phenotype in DMM mice. However, whether it directly inhibits macrophage infiltration or reverses their phenotype warrants further studies.

## 4. Materials and Methods

### 4.1. Materials

Drugs were purchased from several vendors: gallein, TOCRIS biosciences (Bristol, UK); paroxetine hydrocholoride, Calbiochem, Millipore, Burlington, MA, USA; fluoxetine and indomethacin, EMD Millipore, Burlington, MA, USA. Unless otherwise stated, all the reagents used were of analytical grade and were purchased from Sigma-Aldrich (St. Louis, MO, USA).

### 4.2. Study Design

The sample size was determined using the G*Power 3.1 software (3.1.9.7; Heinrich-Heine-Universität Düsseldorf, Düsseldorf, Germany). Based on our previous experience and studies, synovial thickness, the area of uncalcified tibial cartilage, and the total number of chondrocytes are the main parameters affected by DMM. Based on the differences in these parameters between the sham- and DMM-operated mice, we calculated the sample size required for each group to reach 5% significance and 0.80 power. A power analysis showed that at least five mice in each group were required. Thus, we increased the number of mice to 5–10 to account for any unexpected side effects due to surgery or treatments. The end points of 1 week and 12 weeks post-DMM were chosen based on our and others’ experience with the DMM model related to early-stage inflammation peaks [44] and late-stage OA development [16,45,46,47]. The surgeon and lab personnel performing daily drug injections were blinded to the animal identity and treatment. All animals were randomized for treatment. All collected mouse samples were coded and analyzed in a blinded manner until the data were obtained and quantification was completed. The data were then decoded and matched to the corresponding groups, the results were charted, and the statistical analysis was performed. Outlier data, determined by Grubb’s test, were excluded. All studies were performed according to the ARRIVE guidelines 2.0.

### 4.3. Animals

Twelve-week-old male C57BL/6J mice were purchased from The Jackson Laboratories. All mice were housed in groups of three to five mice per micro-isolator cage in a room with a 12 h light/dark schedule. All animal procedures were performed according to the National Institute of Health (NIH) guide for the care and use of laboratory animals and were approved by the Animal Care and Use Committee of the Pennsylvania State University (approval number and date: PRAMS201747946, 21 January 2022).

### 4.4. DMM Surgery

Twelve-week-old male mice were administered DMM surgery to the right knee and sham surgery to the left knee, as described in [46]. Briefly, mice were anesthetized via an intraperitoneal injection of ketamine (60 mg/kg) and xylazine (4 mg/kg), and a 5 mm-long incision was made on the medial side of the knee. Under a dissecting microscope, an incision was made along the medial side of the patellar tendon, opening the joint space. Using a #11 scalpel, the medial meniscotibial ligament (MMTL) was transected, enabling the medial meniscus to move freely. A similar skin incision was made in the sham knees, but the joint structure was not disturbed. For the sham group, both the right and left knees had sham surgeries. After surgery, 4–0 silk sutures were used to close the incision using an interrupted pattern. The mice were provided analgesia via an intraperitoneal injection of buprenorphine (0.5 mg/kg) every 12 h for 72 h, and the sutures were removed after 7 days. The mice were sacrificed at the indicated time points by anesthesia followed by whole-animal perfusion using 10% neutral buffered formalin (NBF). The knees were harvested and fixed for 7 days in 10% NBF, decalcified for 7 days in 10% *w/v* ethylenediaminetetraacetic acid (EDTA), embedded in paraffin, and 5 μm sections were cut and mounted for Safranin-O/Fast Green or IF staining.

### 4.5. Experimental Groups

In 12-week-old male wild-type C57BL/6 mice that received DMM surgeries, vehicle (PBS), paroxetine (5 mg/kg per day), fluoxetine (5 mg/kg per day), gallein (10 mg/kg per day), or indomethacin (2.5 mg/kg per day) were administered daily by intraperitoneal injection. The drug concentrations were selected based on the clinically relevant doses of paroxetine, fluoxetine, and indomethacin and on the minimum effective dose of gallein, as described by us and others [16]. The drug treatments were initiated two days before DMM to achieve Gβγ-GRK2 inhibition at the time of injury and continued for 1 or 12 weeks (early phase and late phase of OA). The mice were euthanized 1 or 12 weeks post-DMM to determine the role of Gβγ-GRK2 signaling in synovitis and OA progression. The knees were collected, formalin-fixed, and paraffin-embedded for further analyses.

### 4.6. Histomorphometry (Safranin-O/Fast Green) Coupled with Histomorphometry Using the Osteomeasure^®^ System

Using Safranin-O/Fast-Green-stained sections, the OsteoMetrics system (OsteoMeasure7 v4.3.0.1, Decatur, GA, USA) was used to quantify the above parameters on three sections from representative levels (50 mm apart) of the medial compartment of the joint for each sample. Live images of the center of the knee joint were collected through an Olympus microscope (10× objective) outfitted with a camera, and a stylus was used to trace the regions of interest (ROIs). A blinded observer quantified the uncalcified articular cartilage area, the total chondrocyte number, the matrix-producing chondrocyte number (cells stained by dark-red color), and the synovial membrane thickness using the built-in area calculation algorithms and quantification functions of the OsteoMeasure^®^ system [47]. Briefly, the total cartilage area was measured by the first line that was drawn across the superior edge of the cartilage surface, where the cartilage meets the joint space. A second line was drawn at the chondro-osseous junction, where the calcified cartilage meets the subchondral bone. The calcified cartilage was determined with a line drawn along the tide mark, which is the naturally occurring line separating the calcified and uncalcified regions of the articular cartilage. A second line was drawn at the chondro-osseous junction, where the calcified cartilage meets the subchondral bone. The uncalcified cartilage area was determined by subtracting the calcified cartilage area from the total area of cartilage. The total chondrocyte number and the matrix-producing chondrocyte number were counted within the uncalcified cartilage region using count functions within the histomorphometry system. The matrix-producing chondrocytes were counted based on presence of Safranin-O staining within the extracellular matrix surrounding the articular chondrocyte, indicating the maintenance of matrix homeostasis by anabolic signaling. Similarly, the anterior femoral synovial thickness was measured using OsteoMeasure^®^ software. The synovial membrane thickness extending from the anterior horn of the medial meniscus to the femur was determined by drawing a line from the inner insertion point on the femur towards the attachment on the meniscus. A second line was drawn from the outer insertion of the femur towards the attachment to the meniscus. The synovial thickness was calculated by dividing the total synovial area by the synovial perimeter [47].

### 4.7. Immunofluorescence (IF) Staining

Paraffin sections were deparaffinized in three changes of xylene for five minutes each, rehydrated in ethanol (two changes of 100% ethanol, followed by two changes of 95% ethanol, for five minutes each, followed by one change of 70% ethanol for two minutes), and rinsed twice in deionized water for one minute each. Antigen retrieval was performed for 10 min at 37 °C using 0.4% pepsin (Sigma P-7000) in 0.1 M hydrochloric acid (HCl) and was followed by permeabilization for 30 min at room temperature using 0.03% Triton X in tris-buffered saline (TBS). The sections were then blocked for 1 h at room temperature in 10% normal goat serum in 1X TBS. The tissue sections were incubated overnight at 4 °C with the specific primary antibody for the target protein (detailed in Table S1). Following three 5 min washes in 1X TBS, the slides were incubated for 1 h at room temperature with a biotinylated secondary antibody (Life Technologies, Waltham, MA, USA), washed again with three changes of 1X TBS, and incubated for 1 h at room temperature with Alexa fluor 647 Streptavidin. The antibody concentrations were determined based on titration experiments for each antibody. Finally, slides were washed with three changes of 1X TBS for 5 min each, followed by one wash in 0.03% Triton X in 1X TBS, and a final 5 min wash in 1X TBS. Mounting and nuclear staining were performed using ProLongTM Gold antifade reagent with DAPI (Invitrogen, Waltham, MA, USA). After staining, imaging was performed using a Zeiss Axio Observer 7 upright wide-field microscope (Carl Zeiss Microscopy GmbH, Jena, Germany) with a uniform exposure time for all the samples within one staining experiment. An image analysis to quantify the percentage of fluorescence area was performed. Using Zen Blue Advanced Image Analysis Software, we quantified the percentage of fluorescence-positive cells: the number of positively stained cells divided by the total number of cells. Then, the fold change relative to the sham group was calculated.

### 4.8. Cell Culture and Treatment

The human monocytes THP1 and synoviocytes (SW982) cell lines were obtained from the American Type Culture Collection (ATCC, Rockville, MD, USA) and were cultured in RPMI-1640 medium supplemented with 10% fetal bovine serum (FBS) and 1% penicillin/streptomycin (Gibco-BRL, Gaithersburg, MD, USA) at 37 °C in a 5% CO_2_ incubator. To determine the direct role of Gβγ-GRK2 signaling in macrophages and FLS inflammatory signaling:

(i) Human THP1 cells were differentiated into resting (MΦ) macrophages (by treating with 100 nM phorbol 12-myristate 13-acetate (PMA) for 72 h). Next, these MΦ macrophages were differentiated into M1 macrophages (by treating with 100 ng/mL lipopolysaccharides (LPS) and 20 ng/mL IFN γ for 48 h) or M2 macrophages (by treating with 100 ng/mL interleukin (IL)-4 for 48 h) [48]. At the time of M1 and M2 macrophage differentiation, we treated cells with phosphate-buffered saline (PBS) (vehicle), 10 µM paroxetine (GRK2 inhibitor), 10 µM gallein (Gβγ inhibitor), 10 µM fluoxetine (SSRI control), or 50 µM indomethacin (anti-inflammatory control) for 48 h.
(ii) SW982 synoviocytes were differentiated into the inflammatory phenotype (FLS) by treating cultures with 5 ng/mL IL-1β for 24 h [49]. At the time of synoviocyte differentiation, we treated with vehicle, paroxetine (10 µM), gallein (10 µM), fluoxetine (10 µM), or indomethacin (50 µM) for 24 h.

### 4.9. RNA Purification and Real Time-Quantitative Polymerase Chain Reaction (RT-qPCR)

mRNA was isolated from human cartilage using the method we published recently [16], yielding RNA integrity number (RIN) values above 7.0 [50]. cDNA was prepared using an Iscript cDNA synthesis kit from Bio-Rad following the manufacturer’s protocol. cDNAs were amplified using TaqMan Gene Expression Assays with the 7500 Fast Real-Time PCR System (Applied Biosystems, Waltham, MA, USA), with GAPDH as the housekeeping gene (Thermo Fisher Scientific, Waltham, MA, USA; described in Table S2).

### 4.10. TUNEL Staining

The apoptosis of articular chondrocytes in cartilage tissues was determined by a TUNEL assay using the FragEL™ DNA Fragmentation Detection kit from EMD Millipore (QIA39-1EA, Billerica, MA, USA). The specimens were visualized under a fluorescence microscope. The number of apoptotic chondrocytes in relation to the total number of cells was quantified in tissue sections.

### 4.11. Statistical Analyses

Multiple responses of various physiological and biochemical assays were analyzed using a one-way ANOVA, as indicated in the mouse studies. The homogeneity of data was tested by running the Brown–Forsythe test, and if the standard deviations were different (*p* ≤ 0.05), the Brown–Forsythe test, Welch’s ANOVA, and an unpaired Welch’s *t*-test were performed; Tukey’s post-hoc analysis was performed if statistical significance (*p* ≤ 0.05) was achieved. All calculations were performed using Graph Pad Prism 9.0.

## 5. Conclusions

We show that early Gβγ-GRK2 inhibition by gallein and paroxetine is of higher therapeutic efficacy in OA. It attenuates OA development by inhibiting the early inflammatory response and by protecting the cartilage.

## Data Availability

The data that support the findings of this study are available from the corresponding author upon reasonable request.

## Supplementary Materials

The following supporting information can be downloaded at: https://www.mdpi.com/article/10.3390/ijms23147933/s1.
