# Supplementary material for: Early Gβγ-GRK2 Inhibition Ameliorates Osteoarthritis Development by Simultaneous Anti-Inflammatory and Chondroprotective Effects
Source: Int J Mol Sci. 2022 Jul 19;23(14):7933. doi: 10.3390/ijms23147933 (PMC9323311; doi:10.3390/ijms23147933)
Supplement: Supplementary file 1 [file ijms-23-07933-s001.zip › Supplementary Tables_07112022.pdf]

Supplementary Tables:

Table S1. List of the primary antibodies (ab, from Abcam; PA, from Thermo Fisher; PIMA5, from Fisher) used for immunofluorescence staining.

Protein Name	Antibody Cat. No
GRK2	ab137666
VCAM1	PA5-86042
CD80	ab64116
CD163	ab182422
iNOS	ab3523
Cytochrome C	PIMA5-11674

Table S2. List of the gene expression assays used for RT-qPCR.

Gene Name	Gene Expression Assay Cat. No
Human TNFα	Hs00174128
Human VCAM1	Hs01003372
Human CD80	Hs01045161
Human CCL22	Hs01574247
Human GAPDH	Hs02758991

Figure S1.

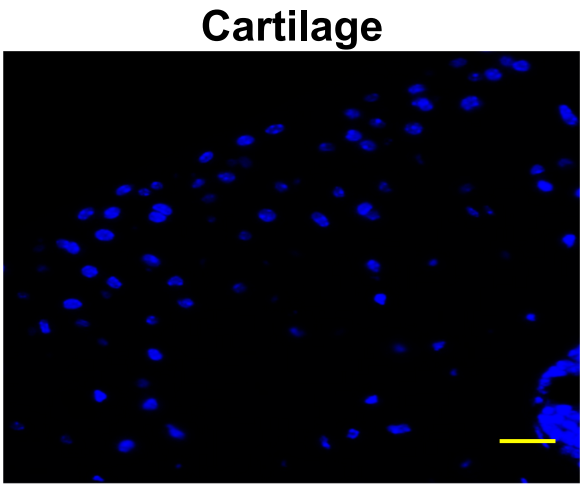

Supplementary Figure S1. Negative controls for IF staining using the corresponding IgG instead of primary antibody. DAPI stains nuclei blue. (20x magnification, scale bar = 10 µm, images acquired with Zeiss Zen Blue 2.6 edition software)
